# Angina Folliculosa of the Pharynx (Hypertrophy of the Follicles of the Pharynx)

**Published:** 1848-09

**Authors:** 


*Angina Folliculosa of the Pharynx*, (hypertrophy of the follicles of the pharynx,) by M. Chomel. (Translated for the Medical Examiner.)

Authors on internal pathology say nothing of this affection; even Pinel does not mention it. It is difficult to account for the silence of so many minute observers, on a disease which, although not very common, is still frequently met with by those who examine the throats of their patients. M. Chomel has collected during the past year, twenty-two cases. It is not a very dangerous disease, but is exceedingly annoying to the patient, who, for that reason, is willing to submit to any treatment.

It is characterized by the abnormal development, or hypertrophy, of the follicles of the pharynx, uvula or soft palate. It may occur at any age, but is mostly to be seen in those from fifteen to thirty years old. It is also more frequent in men than in women, in the proportion of seventeen to five.

We often observe in patients affected with this disease, that there is a very peculiar shape in the superior maxillary bone. Instead of forming a regularly curved arch, it rises very rapidly from the alveolar process of either side, and meets at an acute angle at the top. In consequence of this the nasal fossæ are very much contracted, and the air passes with much more difficulty by that route; the person is obliged, therefore, to breathe more or less with the mouth. The upper lip is usually quite short, exposing the superior incisor teeth to view, and causing the patient to sleep with the mouth open.

We are particular in describing these anatomical peculiarities, says M. Chomel, because we do not regard it as a simple coincidence, but as being concerned in the production of the disease of which we are speaking.

Every organ which secretes more than is natural, tends necessarily to become hypertrophied. In order to prevent the pharynx from becoming dry, in patients with the above conformation, the follicles take on excessive action, and in that way they gradually enlarge.

The patient is often simultaneously affected with *herpes*, while many have suffered with *acne*.

Profession exercises a great influence in the development of the disease in question. In singers we see the follicles very much enlarged sometimes without causing any inconvenience; in that case it cannot be considered a morbid affection. The mucous membrane may even be thickened and red without being diseased. There is no desire to expectorate during the act of singing, but after returning home, or ceasing for a time to sing, secretion takes place freely.

In most cases the disease comes on very gradually, and, after arriving at a certain stage, is very troublesome to the patient. It often begins with a slight cough, accompanied with expectoration just like an ordinary catarrh, which it is supposed will soon get well and requires no attention. When the secretion has become very great, however, it is very troublesome. Some persons say that the attack has been, in them, very sudden.

The disease evinces itself first, by a disagreeable sensation in the back part of the throat; the patient makes painful efforts to swallow or expectorate the mucus lodged at the top of the air passages; he is forced to drink small portions of water to relieve the painful irritation, and does in reality succeed for a short time in this way.

In mild cases, on depressing the tongue with a spoon, several small, round, red points about the size of a millet-seed may be seen. At this stage the follicles are not very prominent, but at a later period they are larger and in the form of disks, in shape like a small lens; sometimes they are ovoid. In other instances, or where the disease is farther advanced, these hypertrophied patches are elongated, and form a projecting tumour, in other cases again, the whole surface of the pharynx is covered with these patches, leaving small points here and there, where the mucous membrane is in its natural condition.

The alteration in the follicles is not always proportionate to the intensity of the symptoms; that depends upon the impressibility of the patient.

It is not unusual to find the surface of the follicles covered with a thick, adherent mucus, which the patient must first remove by coughing or gargling, in order that we may see the extent of the lesion.

The disease is essentially chronic, sometimes it disappears of itself, or yields to the most simple remedies; at others it is stubborn, and resists all the remedies we can use.

The prognosis is not at all grave; it never endangers life, but often obliges the patient to change his profession. The exercise of the voice constantly aggravates the symptoms; the lawyer is obliged to turn magistrate, and the singer to devote himself to instrumental music.

The cough is guttural; it does not come from the chest, neither does the mucus, which is not generally large in quantity; it is in the form of a globule, about the size of a pea, very tenacious and semi-transparent. Occasionally, especially in the morning, the diseased surface is covered with black points, which alarm the patient very much, because he fears phthisis. These points are merely little particles of carbon, which are emitted by the lamps. To the physician they are of no moment.

The voice is changed, and as this is an accompaniment of phthisis, many persons are very much frightened by it, particularly students of medicine; but the remaining symptoms, and the examination of the throat, remove all doubts on this point.

Speech is tiresome, and it is for this reason that the patient commonly comes to consult the physician. Reading aloud is very fatiguing, and the patient cannot continue it long at a time. Sometimes a very tenacious mucus collects, which prevents the person from speaking at all for a short period.

Singers notice that in singing they have lost one or two notes, and their voice is less clear and sweet.

On examining the throat, the mucous membrane covering the palate is found to be redder than usual; this redness is not uniform. There are small red points which increase in number as they approach the uvula; these same red points may be observed on the half-arches of the palate; but it is in the pharynx that the disease is most marked.

The diagnosis is by no means easy; there are individuals, however, whose fauces are so sensitive that it is exceedingly difficult to examine their throats. It is best to frequently repeat the examination, and not to confound the morbid enlargement of the follicles with their natural development which exists in lawyers and singers and demand no treatment.

The treatment is accompanied by many difficulties. Cauterization is the most convenient and the most efficacious, but it is not easy sometimes to limit the caustic in its action, so as not to perforate the mucous membrane. The best caustics are the nitrate of silver, the nitrate of mercury, or nitric acid diluted with three parts of water and applied by means of a sponge. We cauterize, in this way, the whole surface, both healthy and diseased. If the tumours are small, they may be touched several times, at intervals of three or four days. In this way, we get the action both of a caustic and astringent, and sometimes will cure our patient.

The caustics we have just mentioned, should not be used, as a general rule, until milder remedies have failed. We should first have recourse to borax or alum, as a gargle, in solution, (fifteen grains dissolved in a tumbler-full of water;) this will suffice sometimes to restore the parts to their natural condition; mostly, however, it only relieves the patient for the time being.

The sulphur waters of the Pyrenees and Enghier, are frequently employed with success, those of Enghier especially, in consequence, doubtlessly, of the sulphate of lime which they contain. M. Chomel advises to drink two glasses of this water in the morning, gargling with it first, and then swallowing it by mouthfuls, so as to give the parts a sort of local bath. In the summer season this may be combined with baths and douches on the neck. Reading aloud, and talking, must be avoided. The diet must be moderate. All acrid, stimulating food, such as is prepared with butter, or fried, and all vegetables and green fruits should be avoided. Lastly, it is necessary to reduce to powder, or soften such aliment as is very hard—such as crusts of bread—so that it shall not scratch and irritate the fauces when swallowed.—(*Revue Mêdico-Chirurgicale*, from the *Gazette des Hôpitaux.*)

